# Covalent ^18^F-Radiotracers for SNAPTag: A New Toolbox for Reporter Gene Imaging

**DOI:** 10.3390/ph14090897

**Published:** 2021-09-03

**Authors:** Sophie Stotz, Gregory D. Bowden, Jonathan M. Cotton, Bernd J. Pichler, Andreas Maurer

**Affiliations:** 1 Werner Siemens Imaging Center, Department of Preclinical Imaging and Radiopharmacy, Eberhard Karls University, 72076 Tuebingen, Germany; sophie.stotz@med.uni-tuebingen.de (S.S.); gregory.bowden@med.uni-tuebingen.de (G.D.B.); jonathan.cotton@med.uni-tuebingen.de (J.M.C.); bernd.pichler@med.uni-tuebingen.de (B.J.P.); 2 Cluster of Excellence iFIT (EXC 2180) “Image Guided and Functionally Instructed Tumor Therapies”, Eberhard Karls University, 72076 Tuebingen, Germany

**Keywords:** reporter genes, PET imaging, radiotracers, SNAPTag, xenograft

## Abstract

There is a need for versatile in vivo nuclear imaging reporter systems to foster preclinical and clinical research. We explore the applicability of the SNAPTag and novel radiolabeled small-molecule ligands as a versatile reporter gene system for in vivo nuclear imaging. SNAPTag is a high-affinity protein tag used in a variety of biochemical research areas and based on the suicide DNA repair enzyme O^6^-methylguanine methyl transferase (MGMT). Its ligands are well suited for reporter gene imaging as the benzyl guanine core scaffold can be derivatized with fluorescent or radiolabeled moieties for various applications. Three guanine-based SNAPTag ligands ([^18^F]FBBG, [^18^F]*p*FBG and [^18^F]*m*FBG) were synthesized in high yields and were (radio)chemically characterized. HEK293 cells were engineered to express the SNAPTag on the cell surface and served as cell model to assess target affinity by radiotracer uptake assays, Western blotting and SDS-PAGE autoradiography. A subcutaneous HEK293-SNAPTag xenograft model in immunodeficient mice was used for in vivo evaluation of [^18^F]FBBG and [^18^F]*p*FBG while the biodistribution of [^18^F]*m*FBG was characterized in naïve animals. The results were validated by ex vivo biodistribution studies and immunofluorescence staining of the xenografts. All three radiotracers were produced in high radiochemical purity, molar activity and good yields. Western blot analysis revealed successful SNAPTag expression by the transfected HEK293 cells. In vitro testing revealed high target affinity of all three tracers with an up to 191-fold higher signal in the HEK293-SNAPTag cells compared to untransfected cells. This was further supported by a prominent radioactive protein band at the expected size in the SDS-PAGE autoradiograph of cells incubated with [^18^F]FBBG or [^18^F]*p*FBG. The in vivo studies demonstrated high uptake in HEK293-SNAP xenografts compared to HEK293 xenografts with excellent tumor-to-muscle ratios (7.5 ± 4.2 for [^18^F]FBBG and 10.6 ± 6.2 for [^18^F]*p*FBG). In contrast to [^18^F]*p*FBG and its chemical analogue [^18^F]*m*FBG, [^18^F]FBBG showed no signs of unspecific bone uptake and defluorination in vivo. Radiolabeled SNAPTag ligands bear great potential for clinical applications such as in vivo tracking of cell populations, antibody fragments and targeted radiotherapy. With excellent target affinity, good stability, and low non-specific binding, [^18^F]FBBG is a highly promising candidate for further preclinical evaluation.

## 1. Introduction

Metabolic processes that lack a distinct biomarker can be tracked using a reporter that translates a specific action or expression of the process into detectable physical properties like light emission or fluorescence [1]. The incorporation of a reporter gene into the genome leads to the expression of a protein that either exhibits intrinsically detectable properties or can be targeted by specific probes. This general principle has existed for decades and precision reporter systems have been developed for various applications in both basic research and clinical settings [2]. While an array of well-established optical reporter probes are readily available and can be followed on both macroscopic and microscopic scales over long time periods, their clinical use is limited to superficial or invasive applications, due to the high scattering of optical light in tissue [3]. Non-invasive in vivo reporter assays using highly specific radiolabeled ligands are, on the other hand, highly sensitive, not limited by tissue penetration, and can be detected with good temporal and spatial resolution using nuclear imaging techniques like positron emission tomography (PET) and single photon emission computed tomography (SPECT) [4,5,6]. 

In both clinical and preclinical settings, nuclear reporter systems have been used to develop highly target-specific radio-assays, and they have thus become versatile tools for biological research, accurate patient stratification and assessment of therapy response. Several reporter systems are in routine preclinical and clinical use: *Herpes simplex virus* thymidine kinase type 1 (HSV1-tk) or the mutant version HSV1-sr39tk and the radioligand 9-(4-[^18^F]fluoro-3-hydroxymethylbutyl)guanine ([^18^F]FHBG) is a prominent example of a clinically established radioactive reporter system that finds its application in tracking of cell populations and assessment of successful transduction by viral vectors used in gene therapy [7,8,9]. Other important radiolabeled PET reporter systems include *E. coli* dihydrofolate reductase (DHFR) with ^11^C- or ^18^F-labeled trimethoprim (TMP), the sodium iodide symporter (NIS) with [^99m^Tc]TcO_4_^-^ as substrate and the dopamine type 2 receptor (D2R) that can be targeted by 3-(2’-[^18^F]fluoroethyl)spiperone ([^18^F]FESP) and allow for non-invasive, longitudinal and repetitive imaging of the reporter [10,11,12,13,14]. 

Of the many reporter systems that have been investigated, only a few have been successfully translated into routine use. The development of reporter-specific ligands with pharmacodynamic and pharmacokinetic profiles suitable for in vivo use is not a trivial exercise. These probes should ideally be bioorthogonal and not bind to endogenous off-target proteins or compete with endogenous ligands. Moreover, their mechanism of action should allow for their enrichment in reporter expressing tissues though enrichment modes such as metabolic trapping or the irreversible binding to their targets [15].

SNAPTag is a well-established reporter protein originally derived from O^6^-methylguanine methyl transferase (MGMT), an important DNA repair enzyme that dealkylates O^6^-methylguanine by irreversibly transferring the alkyl group to a cysteine in its active center [16]. For its application as a reporter, the binding kinetics were selectively improved upon by targeted mutation within the active site of the enzyme resulting in the faster and more efficient binding of various O^6^-alkylguanine ligands [17,18]. Benzyl substituted O^6^-benzylguanine (BG) derivatives were identified to inhibit the activity of MGMT enzymes faster and more efficiently than O^6^-methylguanine [19]. SNAPTag has several advantages over other reporter gene systems for in vivo imaging: it is of small size and is thus compatible with most gene delivery vectors [20,21]. Its O^6^-BG-based ligands provide a versatile platform for chemical modifications of the enzyme. Moreover, we speculate that its derivation from a human gene might lead to low immunogenicity of the SNAPTag reporter system in clinical settings in contrast to heterologous systems such as bacterial DHFR. 

The high substrate specificity and stability of the protein complex has led to the extensive use of SNAPTag as a tool for live cell imaging [22], as well as for the investigation of protein–protein interactions, optical imaging and targeted drug delivery [23,24]. Radiolabeled BG derivatives have been described but, thus far, not been studied as imaging probes for the SNAPTag reporter, despite their potential advantages for in vivo imaging over established fluorescent BG probes [25,26]. 

**Figure 1:**
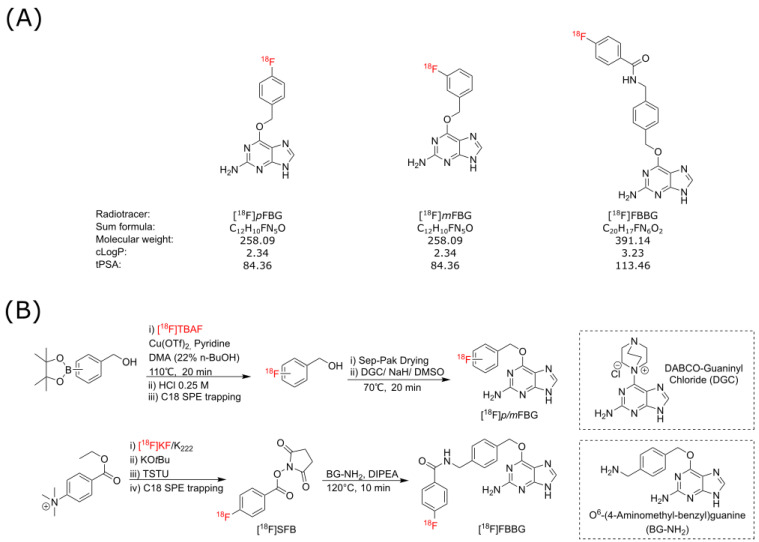
(**A**) Chemical structures and properties of [^18^F]*p*FBG, [^18^F]*m*FBG, and [^18^F]FBBG. (**B**) Schematic representation of the individual radiosyntheses of the three radiotracers. Both [^18^F]*p*FBG and [^18^F]*m*FBG were synthesized from the [^18^F]fluorobenzyl alcohol synthon via the same general radiosynthetic procedure. [^18^F]FBBG was prepared by conjugation of [^18^F]SFB to BG-NH_2_.

As SNAPTag presents as an exciting potential reporter for various novel preclinical and clinical applications, we aimed to establish and evaluate three candidate SNAPTag radiotracers, [^18^F]*p*FBG, [^18^F]*m*FBG and [^18^F]FBBG (Figure 1A), for their binding to a cell surface-bound SNAPTag cell model in vitro and in vivo.

## 2. Results

The three radiotracers discussed in this work were produced from the respective synthons [^18^F]*p/m-*fluorobenzyl alcohol and [^18^F]SFB, via automated synthesis protocols (Figure 1B). [^18^F]*p*FBG was synthesized with an overall activity yield (% AY) of up to 12% (ca. 108 min synthesis time) and decay-corrected radiochemical yield (% RCY) 22 ± 8% (*n* = 3) with excellent radiochemical purity (>95%) and moderate molar activities (41–56 GBq/µmol). [^18^F]*m*FBG was synthesized in up to 21% AY (ca. 103 min synthesis time) and 44 ± 13% RCY (*n* = 3) with >95% radiochemical purity in comparable molar activities. [^18^F]FBBG was synthesized with up to 11% AY, 18% RCY, >95% radiochemical purity and 25–107 GBq/µmol molar activity. Stability of the three radiotracers was assessed in mouse serum over a time span of 240 min and the chromatographs did not reveal any radiometabolites (Figure S2).

**Figure 2:**
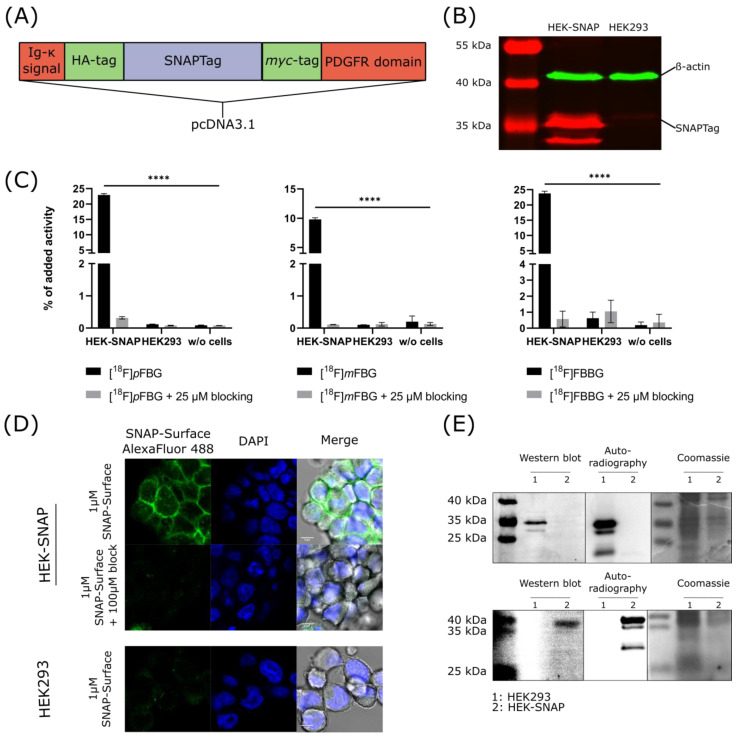
In vitro characterization of the HEK-SNAP cells. (**A**) Schematic depiction of the construct expressed by the HEK-SNAP cells. (**B**) Western blot of HEK-SNAP cell lysate. The HEK-SNAP cells show a band at the expected size of the SNAPTag (anti-*myc* antibody, 33 kDa, red) while HEK293 control cells show no band at this size. β-Actin was used as loading control. (**C**) Cell uptake experiments with [^18^F]*p*FBG, [^18^F]*m*FBG and [^18^F]FBBG. All radiotracers revealed high accumulation in HEK-SNAP cells whereas the control cells only show background uptake. (**D**) Fluorescence microscopy images of HEK-SNAP cells and HEK293 control cells that confirm the expression of the SNAPTag on the cell surface. (**E**) SDS-PAGE autoradiographs of cell lysates incubated with either [^18^F]*p*FBG (upper image) or [^18^F]FBBG (lower image) showing the SNAPTag at the expected size with validation by Western blot and Coomassie staining as loading control.

HEK293 cells were successfully transfected with the desired construct (Figure 2A) as confirmed by Western blotting with an anti-*myc* antibody (Figure 2B). The expression of the SNAPTag on the cell surface was verified by fluorescence microscopy of transfected cells incubated with a commercial SNAPTag ligand. Specificity was ensured in a control experiment where the binding of the probe was blocked using the non-fluorescent ligand (Figure 2D). [^18^F]*p*FBG, [^18^F]*m*FBG and [^18^F]FBBG cell uptake experiments demonstrated significantly higher accumulation of the respective radiotracer in HEK-SNAP cells compared to control cells (191.3-fold, 97.9-fold and 38.3-fold higher, respectively, for [^18^F]*p*FBG, [^18^F]*m*FBG and [^18^F]FBBG, *p* < 0.0001). Tracer uptake was blockable to baseline by co-incubation with the corresponding non-radioactive ligand (Figure 2C). Additionally, SDS-PAGE autoradiography was performed with lysates from cells incubated with [^18^F]*p*FBG or [^18^F]FBBG and revealed a radioactive band at the respective size of the SNAPTag as further confirmed by Western blotting (Figure 2E).

**Figure 3:**
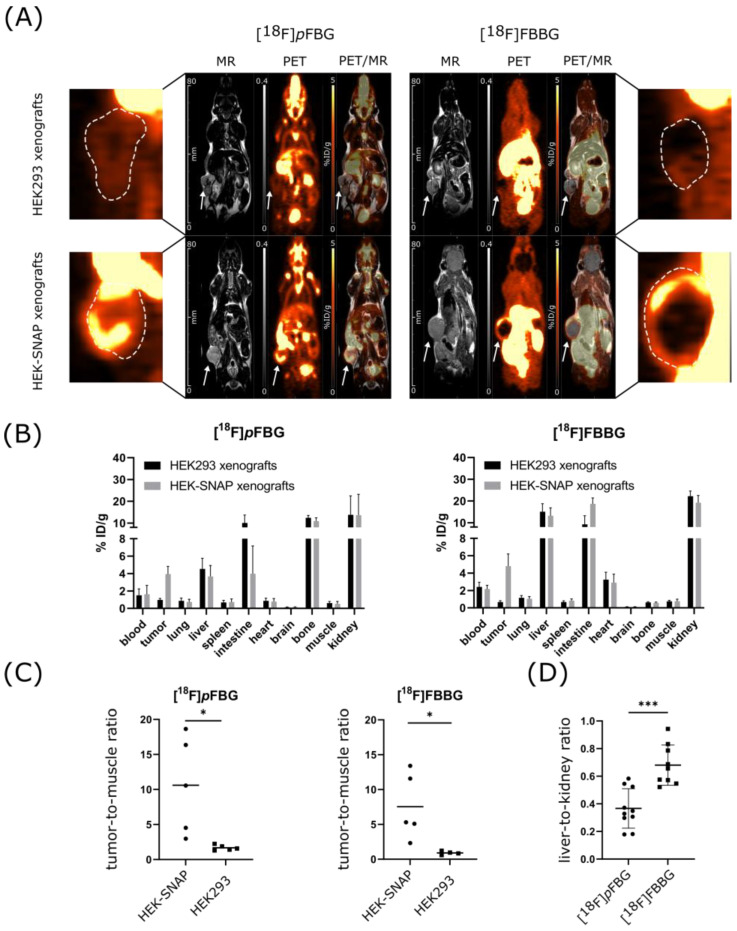
In vivo evaluation of [^18^F]*p*FBG and [^18^F]FBBG. (**A**) PET and MR images of representative mice with HEK-SNAP xenografts (upper images) or HEK293 control xenografts (lower images). Xenograft are indicated with white arrows and presented enlarged on the sides, outlined with a white dashed line. Images were taken after 1h of resting uptake. (**B**) Ex vivo biodistribution analysis by gamma-counting of [^18^F]*p*FBG (*n* = 5) and [^18^F]FBBG (*n* = 5 for HEK-SNAP, *n* = 4 for HEK293). (**C**) TMRs of HEK-SNAP and HEK293 xenografts imaged with either [^18^F]*p*FBG (*n* = 5 per group) or [^18^F]FBBG (*n* = 5 for HEK-SNAP and 4 for HEK293). (**D**) Liver-to-kidney ratios of [^18^F]*p*FBG (*n* = 10) and [^18^F]FBBG (*n* = 9). * *p* < 0.05, *** *p* < 0.001.

**Figure 4:**
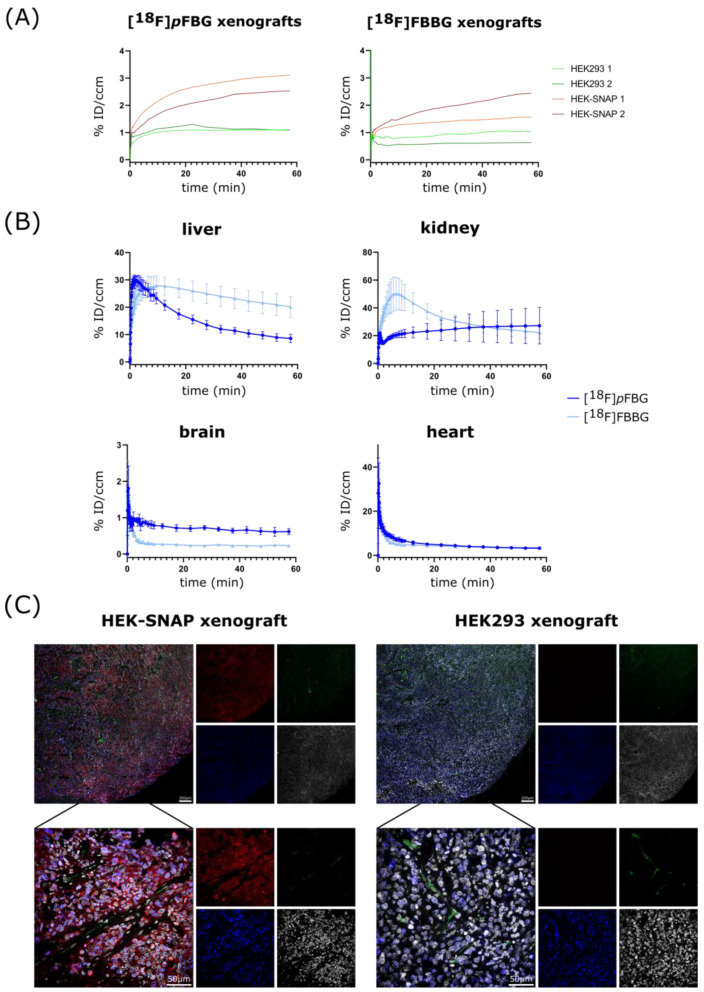
Pharmacokinetic evaluation of [^18^F]*p*FBG and [^18^F]FBBG, and ex vivo immunofluorescence. (**A**) TACs of the individual xenografts. HEK-SNAP xenografts are plotted in red and HEK293 control xenografts in green. (**B**) TACs of liver, kidney brain and heart for [^18^F]*p*FBG (dark blue) and [^18^F]FBBG (light blue) (*n* = 4). (**C**) Representative immunofluorescence images of HEK293 xenograft tissue and HEK-SNAP xenograft tissue. CD31 is stained in green, Ki67 blue, nuclei are displayed in grey and the SNAP protein was visualized by an anti-*myc*-tag antibody shown in red.

PET and MR imaging of HEK-SNAP or HEK293 xenograft-bearing mice revealed high abdominal uptake and both renal and hepatobiliary clearance pathways for all three radiotracers, while [^18^F]*p*FBG and [^18^F]*m*FBG additionally presented high bone uptake after administration (Figure 3A and Figure S3A). The ex vivo biodistribution analysis verified the observed high abdominal uptake and showed radiotracer accumulation mainly in liver, kidney and intestine for all three radiotracers. The radioactivity detected in the bones was also reflected in the ex vivo biodistribution analysis (Figure 3B and Figure S3B). To further assess the three radiotracers’ pharmacokinetic properties, the time activity curves (TACs) of liver, kidney, brain and heart were obtained ( Figure 4B and Figure S2 for [^18^F]*m*FBG). [^18^F]*p*FBG showed a faster liver clearance and lower initial kidney uptake, confirmed by a significantly lower ex vivo liver-to-kidney ratio (0.37 vs. 0.68, *p* = 0.0002, Figure 3D). Both [^18^F]*p*FBG and [^18^F]FBBG exhibited low accumulation in the brain. Both radiotracers showed fast blood clearance with a calculated blood half-life of 3.5 and 1 min, respectively, for [^18^F]*p*FBG and [^18^F]FBBG.

HEK-SNAP xenografts could be clearly differentiated from the control group xenografts by the higher radiotracer accumulation in the PET images and ex vivo biodistribution analysis. Both [^18^F]*p*FBG and [^18^F]FBBG showed a significantly higher tumor-to-muscle ratio (TMR) in comparison to non-transfected HEK293 xenografts (10.6 vs. 1.7, *p* = 0.02 and 7.5 vs. 0.9, *p* = 0.03, respectively, Figure 3C). In line with our hypothesis, the resulting TACs revealed a higher overall activity uptake of the HEK-SNAP xenografts in comparison to HEK293 control xenografts for both radiotracers (Figure 4A). In this model, [^18^F]*p*FBG tended towards a higher absolute accumulation in the xenografts compared to [^18^F]FBBG. 

Ex vivo immunofluorescence staining revealed no detectable SNAPTag expression in HEK293 xenograft tissue in contrast to strong signal (*myc* tag, red) in HEK-SNAP xenograft tissue (Figure 4C). Notably, the expression of the SNAPTag fusion protein decreases towards the xenograft core, along with the density and size of blood vessels (CD31, green), alluding to the beginning of tumor necrosis. This was also observable in the PET images where the radiotracers accumulated mainly in the periphery of the xenografts.

## 3. Discussion

Nuclear reporter gene systems are versatile tools for biological and medical research. SNAPTag is a reporter gene that is highly popular in microscopic research and in vivo imaging of optical probes. Although PET imaging is a superior modality for quantitative and sensitive tomographic imaging, no PET radiotracers have so far been described for this reporter. 

SNAPTag possesses almost ideal properties as a nuclear imaging reporter system in preclinical research that could eventually also be translated for application in human patients: it binds its ligands irreversibly and covalently, is of relatively small size (20 kDa), and is not expressed endogenously, which reduces binding in untargeted tissues. A significant advantage of SNAPTag over other well established PET reporter systems, such as the combination of [^18^F]FHBG and HSV1-tk or HSV1-sr39tk, is that the O^6^-benzylguanine ligands can be easily chemically modified for adaptation to different modalities or fine-tuned in terms of their pharmacological properties. This could include the introduction of fluorophores for optical imaging or microscopy, ^18^F for PET imaging, or conjugation to a chelator for radiometal labeling for SPECT or PET imaging or radiotherapy. 

We designed three SNAPTag radiotracers ([^18^F]*p*FBG, [^18^F]*m*FBG, and [^18^F]FBBG) that share the common BG pharmacophore for targeting the enzyme but differ in their radiolabeling strategy and thus in their size and polarity. [^18^F]*p*FBG and [^18^F]*m*FBG were conceived with minimalism in mind; to have the simplest fluorinated BG derivative possible. [^18^F]FBBG was built off a derivatizable and commercially available core structure. This allowed us to identify the best tracer candidate for future applications. 

[^18^F]FBBG was generated by conjugation of [^18^F]fluorobenzoate to an amine-functionalized commercially available BG ligand. [^18^F]*p*FBG and [^18^F]*m*FBG were generated by the radiolabeling of fluorobenzyl alcohol and subsequent conjugation with the guanosine system. The latter leads to two highly similar molecules that only differ in the position of the ^18^F atom in the aromatic ring (*meta* or *para*). Only [^18^F]*p*FBG and [^18^F]FBBG were fully evaluated in xenograft mouse models while [^18^F]*m*FBG was only evaluated in naïve animals in order to investigate whether it shows similarly high bone uptake as [^18^F]*p*FBG. All tracers underwent stability assays which confirmed that they are stable in murine serum under the conditions expected in in vitro and in vivo experiments. 

For the generation of a genetically engineered cell model that would enable in vitro and in vivo evaluation of the radiotracers, we fused the SNAP26f sequence with a transmembrane domain and an upstream Igκ signal peptide sequence to enable cell surface expression. Additionally, we incorporated the myc-tag in the sequence for in vitro Western blot and ex vivo immunofluorescence detection of the transgene. This plasma membrane-tethered design ensured that the SNAP enzyme was easily accessible to candidate ligands at the cell surface, aiding in vitro and in vivo evaluation. Such surface-expressed SNAPTag moiety potentially enables the use of a variety of bigger or more polar ligands, as they do not need to cross the cell membrane.

With the cell model and the radiotracers in hand, the in vitro evaluation indeed revealed the anticipated fast and specific uptake of all three radiotracers in the SNAPTag expressing cells compared to control cells. SDS-PAGE autoradiography confirmed the absence of undesired off-target binding as only a single band corresponding to the expected molecular size was visible.

The promising in vitro results encouraged subsequent in vivo studies for assessment of xenograft uptake and pharmacokinetics of the ligands [^18^F]*p*FBG and [^18^F]FBBG in a pilot study in mice bearing SNAPTag-expressing xenografts. Indeed, both [^18^F]*p*FBG and [^18^F]FBBG demonstrated excellent xenograft uptake with very low endogenous background uptake in non-transfected tumors. Immunofluorescence microscopy revealed a loss of SNAPTag expression in the xenograft core that correlated with a lower expression of the proliferation marker Ki67, which could be caused by the relatively large xenograft size. The high abdominal uptake due to the excretion of the radiotracers implies that optimal contrast can be achieved within extra-abdominal tissues. Additionally, the radiotracers seem not to cross the blood–brain barrier (BBB), limiting their use for brain-related applications in individuals where the BBB has not been compromised. [^18^F]*p*FBG exhibited a significantly lower (*p* = 0.0002) liver-to-kidney ratio after 1h compared to [^18^F]FBBG (Figure 3D), driven by the rapid clearance from the liver in accordance with the TACs (Figure 4B). However, [^18^F]*p*FBG suffers from rapid defluorination of the molecule in vivo despite appearing stable in serum. Changing the position of the radiolabel ([^18^F]*m*FBG) did not result in the improvement of metabolic stability when tested in naïve animals, limiting the application of both FBG radiotracers in mouse models. However, since murine metabolism differs drastically from human metabolism the relevance of this phenomenon for potential clinical applications has to be addressed in future studies. Additional chemical modifications of [^18^F]*p*FBG addressed in future work might result in increased metabolic stability [27].

We have demonstrated in this work that the SNAPTag reporter protein in combination with radiolabeled O^6^-benzylguanine ligand derivatives represent a promising tool that fulfills requirements for a preclinically applicable and clinically translatable reporter gene system. Despite [^18^F]*p*FBG featuring higher xenograft and lower liver uptake, [^18^F]FBBG is favored for further preclinical evaluation as it shows no signs of defluorination and can be readily synthesized using the widely accessible radiochemical synthon [^18^F]SFB and a commercially available BG intermediate.

## 4. Materials and Methods

### 4.1. General Radiochemistry

[^18^F]Fluoride was produced on a medical cyclotron (PETtrace 800, GE Healthcare, Uppsala, Sweden) using the ^18^O(p,n)^18^F nuclear reaction. [^18^F]FBBG synthesis was automated on a modified TRACERlab radiochemical synthesizer (GE Healthcare) while [^18^F]*p*FBG and [^18^F]*m*FBG were synthesized on a Elixys FLEX/CHEM radiochemical synthesizer equipped with a PURE/FORM purification module (Sofie Bioscience, Dulles, VA, USA). The high-performance liquid chromatography (HPLC) signal at 254 nm (Infinity 1260, Agilent, Waldbronn, Germany) was used for calculation of the carrier concentration from calibration curves. HPLC columns were purchased from Phenomenex (Aschaffenburg, Germany). The boropinacolate precursors for radiosynthesis of [^18^F]fluorobenzyl alcohols were synthesized as previously described. The preparation and characterization of the other radiolabeling precursors and the corresponding non-radioactive standards is described in detail in the supplementary information. Chemdraw 19.1 (PerkinElmer, Waltham, MA, USA) was used for representation of chemical structures and calculation of chemical properties.

### 4.2. [^18^F]pFBG and [^18^F]mFBG Synthesis

[^18^F]*p*FBG and [^18^F]*m*FBG were synthesized via a fully automated two-step process [28]. The first step featured a DoE optimized copper-mediated radiofluorination (CMRF) of either 4-(hydroxymethyl)phenylboronic acid pinacol ester or 3-(hydroxymethyl)phenylboronic acid to the respective *para-* or *meta-*fluorobenzyl alcohol. The [^18^F]fluoride was processed to [^18^F]TBAF in accordance to the previously published procedure [29]. The processed [^18^F]TBAF was reacted with the relevant precursor (19 µmol) in the presence of Cu(OTf)_2_ (5 µmol) and pyridine (25 µmol) in dimethyl acetamide (DMA) with *n*-BuOH (22%) as a co-solvent (total volume = 700 µL). Air (30 mL) was introduced to the reactor, and the reaction was then warmed to 120 °C. After 20 min, the reaction mixture was quenched with HCl (0.25 M, 4 mL) and the product was trapped out of the solution using a C18 solid phase exchange (Sep-Pak C18 Plus Light, Waters, Waltham, MA, USA) cartridge with an aluminum oxide N cartridge (Sep-Pak Alumina N Plus Light, Waters) stacked on top. The resulting fluorobenzyl alcohol was eluted from the cartridge stack with diethyl ether (2 × 1.5 mL) over a Sep-Pak Dry cartridge (Na_2_SO_4_) and into the second reactor containing DABCO-guaninyl chloride (DGC, 50 µmol), sodium hydride (60% in mineral oil, 20–25 mg), and dimethyl sulfoxide (DMSO, 600 µL). The diethyl ether was evaporated from the biphasic mixture and the remaining solution was reacted at 70 °C for 20 min. The reaction was quenched with the addition of HPLC buffer (ammonium formate 25 mM, 4 mL) and was subsequently purified with semipreparative HPLC (Supelcosil ABZ+; 5 µm; 250 × 10 mm, 30% acetonitrile in ammonium formate buffer 25 mM, 6 mL/min). After collection of the product radioactive fraction in a water dilution reservoir (60 mL), the product was trapped on a hydrophilic lipophilic balance (HLB, Waters) cartridge, eluted with ethanol (0.5 mL), and formulated for injection with phosphate buffered saline (PBS, 4.5 mL).

### 4.3. [^18^F]FBBG Synthesis

[^18^F]Fluoride was trapped on an ion exchange cartridge (Sep-Pak Plus Light QMA Carb) preconditioned with 10 mL 1 M NaHCO_3_ and 10 mL H_2_O. [^18^F]Fluoride was eluted with 2 mL 4% H_2_O in MeCN containing 9.5 mg Kryptofix 2.2.2 and 1.7 mg K_2_CO_3_ and azeotropically dried under a stream of helium. The synthon *N*-succinimidyl 4-[^18^F]fluorobenzoate ([^18^F]SFB) was synthesized in a three-step one-pot reaction. Successively, 5 mg ethyl-4-trimethylammonium benzoate triflate (SFB precursor) in 0.5 mL dimethylsulfoxide (DMSO) (8 min at 120 °C), 15 mg *t*BuOK in 0.5 mL DMSO (5 min at 120 °C) and 30 mg *N,N,N′,N′*-tetramethyl-*O*-(*N*-succinimidyl)uronium tetrafluoroborate (TSTU) in 2 mL MeCN (5 min at 100 °C) were added to the reaction vial. After cooling to 50 °C and addition of 1 mL 5% acetic acid to prevent hydrolysis, the reaction was diluted in 25 mL H_2_O and trapped on a Sep-Pak Plus Light C18 cartridge (Waters) preconditioned with 10 mL ethanol and 10 mL H_2_O. [^18^F]SFB was eluted from the cartridge back into the reactor with a solution containing 6-((4-(aminomethyl)benzyl)oxy)-7*H*-purin-2-amine (BG-NH_2_, 10 mg, 37 μmol, AA Blocks, San Diego, CA, USA), Hünig’s base (33 μL, 185 μmol) in dimethyl formamide (DMF) (1 mL). After heating to 120 °C for 10 min, the reactor was cooled to room temperature. HPLC eluent (2.0 mL of 25% MeCN in 0.1% aqueous trifluoroacetic acid) was added before semipreparative HPLC purification (Luna C18(2), 5 µm, 100 Å, 250 × 10 mm; 7 mL/min). The final product was collected into a dilution reservoir containing 20 mL water and the resulting solution was passed over a conditioned Oasis HLB Plus Light cartridge (Waters), after which the product was eluted with 0.5 mL EtOH and diluted with 4.5 mL PBS.

### 4.4. Cell Culture and Generation of HEK-SNAP Cells

The coding sequence for cell surface expression of SNAPTag was constructed by in silico insertion of the sequence SNAP26f downstream of the *Bgl*II site of the pDisplay (Thermo Fisher, Waltham, MA, USA) multiple cloning site sequence. The resulting open reading frame including the Kozak sequence was synthesized by BioCat (Heidelberg, Germany) and inserted in between the *Xho*I and *Xba*I sites of pcDNA3.1(+) (Thermo Fisher). A scheme of the open reading frame is depicted in Figure 2A and the sequence is shown in Figure S1. 

HEK293 cells were purchased from Cell Line Service (Eppelheim, Germany) and cultured in Dulbeccos’s modified Eagle’s medium (DMEM) supplemented with 10% fetal calf serum (FCS), 100 U/mL penicillin and 100 µg/mL streptomycin. Cells were stably transfected with the pcDNA3.1 vector containing the SNAPTag sequence using Lipofectamine 3000 according to the manufacturer’s protocol and selection with G418 (500 µg/mL, Biochrom, Berlin, Germany). Single clones were isolated using limiting dilution and tested for protein expression by Western blot. 

### 4.5. Western Blot

Cells were lysed using radioimmunoprecipitation assay (RIPA) buffer (Thermo Fisher) containing protease inhibitor (cOmplete Mini, EDTA-free, Roche, Basel, Switzerland) and the protein concentration was determined using a commercial bicinchoninic acid (BCA) assay kit (Thermo Fisher). Samples containing 80 µg protein were denatured in reducing sample loading buffer and subjected to discontinuous SDS-PAGE on gels containing 12% polyacrylamide. After completion of the electrophoresis run, the proteins were transferred onto a polyvinylidene fluoride membrane using the Mini-PROTEAN Tetra system (Bio-Rad, Hercules, CA, USA) and blocked for 1 h with Odyssey blocking buffer (Li-Cor, Lincoln, NE, USA). The blot was incubated at 4 °C overnight in phosphate buffered saline (PBS) with primary antibodies (mouse anti-*myc*, clone 9E10, 2 µg in 10 mL and rabbit anti-β-actin, clone C4, Sigma-Aldrich, 1:3000). After washing twice with PBS-T for 10 min, the membrane was incubated for 1 h in PBS with secondary antibodies (goat anti-mouse IgG, IRDye 680 RD, 1:7000 and goat anti-rabbit IgG, IRDye 800 CW, 1:7000, Li-Cor), washed again twice with PBS-T and subsequently imaged with an Odyssey Sa Infrared Imaging System (Li-Cor).

### 4.6. Fluorescence Microscopy

A total of 0.1 × 10^6^ cells were seeded in a 4-chamber culture slide (BD Falcon, Franklin Lakes, NJ, USA) the day before the experiment to achieve 50% confluency. Cells were then incubated for 30 min at 37 °C with 250 µL of medium containing either 1 µM of a commercial fluorescent SNAPTag ligand (SNAP-Surface AlexaFluor 488, NEB, Ipswich, MA, USA), or 1 µM of fluorescent SNAPTag ligand with 100 µM of the non-fluorescent compound (*p*FBG standard, blocking control). DAPI solution (250 µL, 1:12,000 in PBS) was added for 5 min after removal of the staining solution. The cells were washed twice with 500 µL PBS, fixed with 500 µL 4% paraformaldehyde solution for 30 min and mounted with quick-hardening mounting medium (Eukitt, Sigma-Aldrich, St. Louis, MO, USA). Fluorescence microscopy was performed using a Zeiss LSM 710 ConfoCor3 microscope (Carl Zeiss, Jena, Germany) with a C-Apochromat × 40 N.A. 1.2 water immersion objective (Zeiss) and argon ion (488 nm) and DPSS (561 nm) excitation lasers.

### 4.7. SDS-PAGE Autoradiography

A total of 1 × 10^6^ cells were incubated with 3 MBq of [^18^F]*p*FBG or [^18^F]FBBG in 250 µL medium for 15 min at 37 °C (or medium only for control). After washing with 1 mL of complete medium, 200 µL RIPA buffer with protease inhibitor were added and the samples were incubated for 10 min at room temperature. A total of 20 µL of sample were mixed with 4 µL 6× reducing loading buffer, heated to 95 °C for 5 min and SDS-PAGE was performed as described above. A storage phosphor screen (Molecular Dynamics, Caesarea, Israel) was exposed to the gel in a light-shielded cassette for approximately 10 half-lives of ^18^F (18 h), and the screen was scanned using a phosphor imager (Storm 840, Molecular Dynamics). As a loading control, a part of the gel was stained with a commercial Coomassie solution (InstantBlue Protein Stain, Expedeon/Biozol, Eching, Germany) according to the manufacturer’s instruction.

### 4.8. In Vitro Tracer Uptake Experiments

A total of 1 × 10^6^ cells in 0.9 mL medium were dispensed into gamma counter tubes (quadruplicates) and 0.6 mL tracer solution (2 MBq/mL in complete medium, either with 2.5 µL/mL DMSO as vehicle control or 2.5 µL/mL 10 mM non-radioactive standard (25 µM), blocking group) were added. After incubation for 30 min at 37 °C, cells were washed twice with 0.5 mL and once with 1.5 mL complete medium. The supernatant was removed after a final centrifugation step, the samples were measured in a gamma counter (WIZARD2, PerkinElmer, Waltham, MA, USA) and uptake was quantified as percent of added activity.

### 4.9. Serum Stability

Serum stability was assessed by mixing [^18^F]*p*FBG, [^18^F]*m*FBG or [^18^F]FBBG solution 1:1 with C57BL/6J mouse serum. After 0, 30, 60, 120 and 240 min incubation at 37 °C, samples were drawn and the proteins were precipitated by adding ice-cold MeCN to a final concentration of 50%. The supernatant after centrifugation (12,100× *g*, 90 s) was analyzed by HPLC as described for radiotracer quality control.

### 4.10. PET/MR Imaging and Ex Vivo Biodistribution Analysis

All animal experiments were performed in accordance with the German animal welfare act and approved by the local authorities (Regierungspräsidium Tuebingen, R3/18). Mice were purchased from Charles River Laboratories (Sulzbach, Germany) and kept under 1.5% isoflurane evaporated in oxygen (1.5 L/min) as anesthetic during the experiments. Female NOD.CB17-Prkdc^scid^/J mice (7 weeks old) were subcutaneously injected with either 5 × 10^6^ HEK293 cells or HEK-SNAP cells in the right flank (*n* = 5 per xenograft group). After tumors reached a suitable size (492.3 mm^3^ ± 438.3 mm^3^), either 1 h dynamic scans (*n* = 2 per group) or 10 min static scans after 1 h resting uptake (*n* = 3 per group) were performed on an Inveon dedicated microPET system (Inveon D-PET, Siemens, Knoxville, TN, USA) with 12.6 ± 0.2 MBq [^18^F]*p*FBG or 12.3 ± 1 MBq [^18^F]FBBG injected intravenously per mouse. Naïve animals were injected with 12.4 ± 0.3 MBq [^18^F]*m*FBG intravenously per mouse and imaged dynamically for 1 h (*n* = 2) or statically after 1 h resting uptake (*n* = 1). Subsequently, an anatomical MR scan with a 7 Tesla BioSpec 70/30 USR (Bruker Biospin MRI GmbH) using a T2-weighted 3-dimensional turbo spin-echo sequence was performed after which the mice were sacrificed by cervical dislocation. The organs were collected, weighed and the radioactivity was measured in a gamma counter (WIZARD2, PerkinElmer, Waltham, MA, USA). Reconstruction and analysis of the acquired PET/MR data was performed with Inveon Acquisition Workplace and Inveon Research Workplace, respectively, using dynamic framing and an ordered subset expectation maximization (OSEM) 3D algorithm. Regions of interest (ROIs) were drawn in accordance with the acquired MR images and co-registered with the PET data to obtain time-activity curves (TACs). 

### 4.11. Ex Vivo Immunofluorescence

Immunofluorescence staining was performed by the Department of Dermatology at the University Hospital Tuebingen, Germany. Sections of paraffin-embedded xenografts were blocked with donkey serum for 30 min and incubated with primary antibodies overnight (rat anti-CD31, 1:100, DIA-310, Dianova, Hamburg, Germany; mouse anti-c-myc, clone 9E10 hybridoma supernatant 1:20; rabbit anti-Ki67, 1:100, ab15580, Abcam, Cambridge, UK). After washing, the sections were incubated for 1 h at room temperature with secondary antibodies (donkey anti-rat IgG Alexa488, 1:125, 712-546-153 Dianova; donkey anti-mouse IgG Cy3, 1:125, 715-166-151 Dianova; donkey anti-rabbit IgG, 1:125, 711-606-152 Dianova). Nuclei were stained with DAPI solution (D9542, Sigma-Aldrich) for 30 min, the samples were subsequently mounted with Mowiol (Sigma-Aldrich) and imaged on an LSM 800 microscope (Carl Zeiss).

### 4.12. Statistical Analyses

Statistical analyses are represented as mean value ± standard deviation. Analyses were performed in GraphPad Prism 9 using nonparametric t-tests and *p*-values < 0.05 were considered statistically significant (*: *p*-value < 0.05; **: *p*-value < 0.01; ***: *p*-value < 0.001; ****: *p*-value < 0.0001).

## 5. Conclusions

In this proof-of-principle study, we successfully targeted SNAPTag with radiolabeled BG derivatives for in vivo PET imaging. Tumors expressing the SNAPTag were clearly distinguishable from control tumors in the in vivo images and ex vivo biodistribution data. Our results indicate that SNAPTag in combination with our novel radiotracers will be a powerful toolbox for future preclinical and clinical challenges. The option to combine PET with other imaging modalities targeting the same reporter opens new avenues for multimodal and multiscale tracking of genetic vectors and cell populations. 

## Data Availability

Data is contained within the article and supplementary materials.

## Supplementary Materials

The following are available online at https://www.mdpi.com/article/10.3390/ph14090897/s1, Figure S1: Sequence of the pcDNA 3.1 plasmid insert containing the SNAPTag sequence. Figure S2: Serum stability of the three radiotracers. Figure S3: In vivo evaluation of [^18^F]mFBG in naïve animals. Figure S4: TACs of liver, kidney, brain and heart for [^18^F]*m*FBG.
