# Supplementary material for: Covalent 18F-Radiotracers for SNAPTag: A New Toolbox for Reporter Gene Imaging
Source: Pharmaceuticals (Basel). 2021 Sep 3;14(9):897. doi: 10.3390/ph14090897 (PMC8466261; doi:10.3390/ph14090897)
Supplement: Supplementary file 1 [file pharmaceuticals-14-00897-s001.zip › pharmaceuticals-1356693-supplementary.pdf]

Supplementary Information

for

Covalent ^{18}F-Radiotracers for SNAPTag: a New Toolbox for Reporter Gene Imaging

Sophie Stotz*[1,2], Gregory D. Bowden*[1,2], Jonathan M. Cotton[1,2], Bernd J. Pichler[1,2] and Andreas Maurer[1,2]

[1]Werner Siemens Imaging Center, Department of Preclinical Imaging and Radiopharmacy, Eberhard Karls University, Tuebingen, Germany

[2]Cluster of Excellence iFIT (EXC 2180) "Image Guided and Functionally Instructed Tumor Therapies", Eberhard Karls University, Tuebingen, Germany

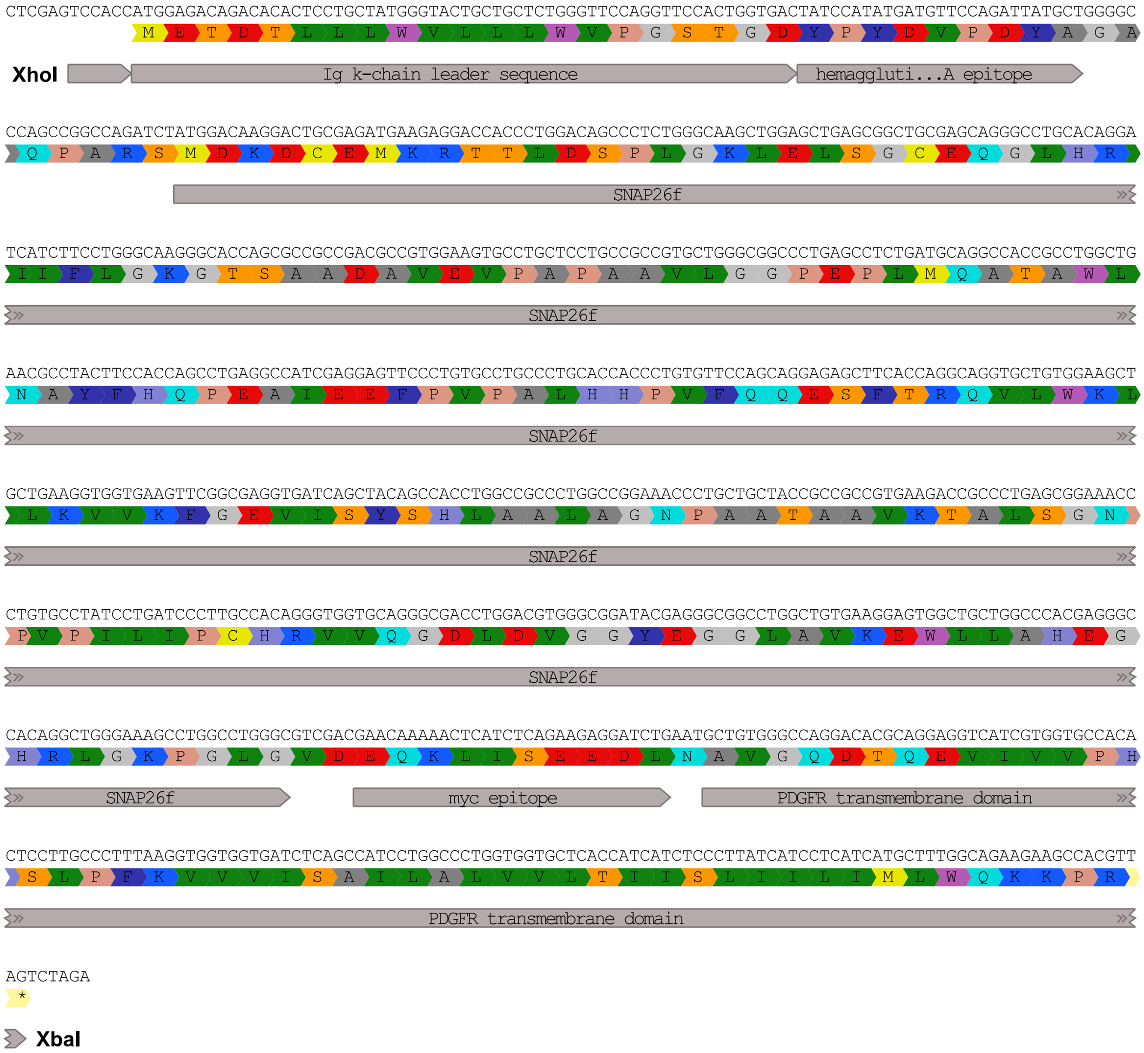

Supplementary Figure S1: Sequence of the pcDNA 3.1 plasmid insert containing the SNAPTag sequence.

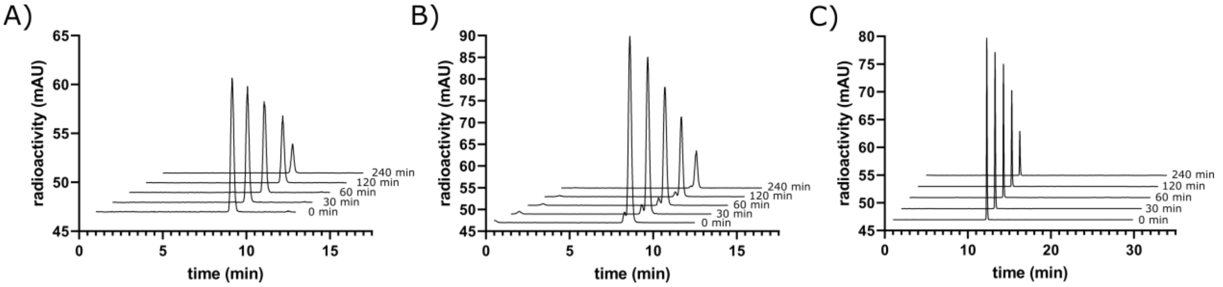

Supplementary Figure S2: Serum stability of the three radiotracers. A) [^{18}F]*p*FBG, **B)** [^{18}F]*m*FBG, **C)** [^{18}F]FBBG. HPLC samples were drawn after 0, 30, 60, 120, and 240 min.

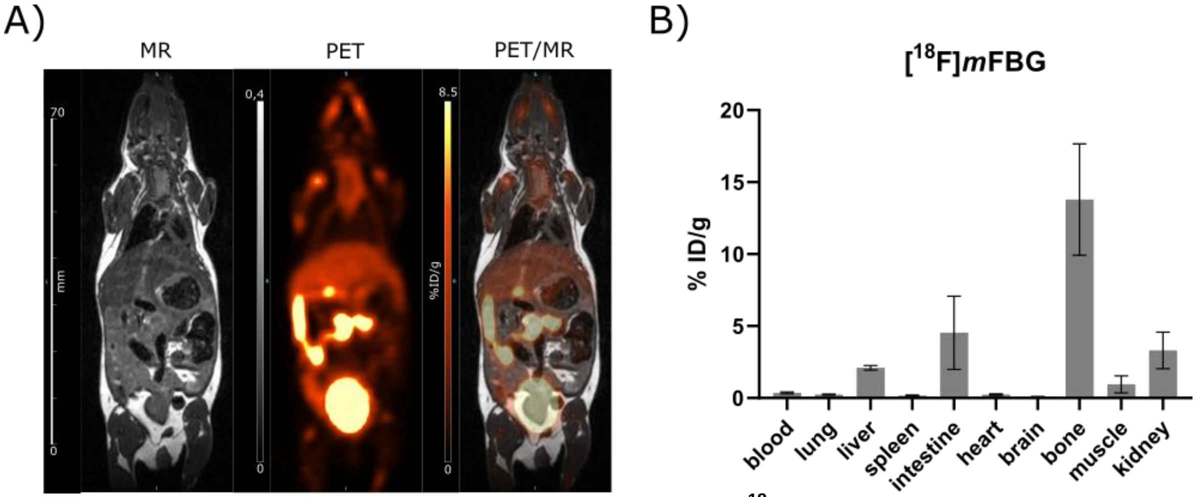

Supplementary Figure S3: ***In vivo*** **evaluation of [^{18}F]*m*FBG in naïve animals. A)** Representative PET and MR images of a mouse injected with [^{18}F]*m*FBG. Images were generated from the last 10 min of a 1 h dynamic scan. **B)** *Ex vivo* biodistribution analysis of [^{18}F]*m*FBG (n=3).

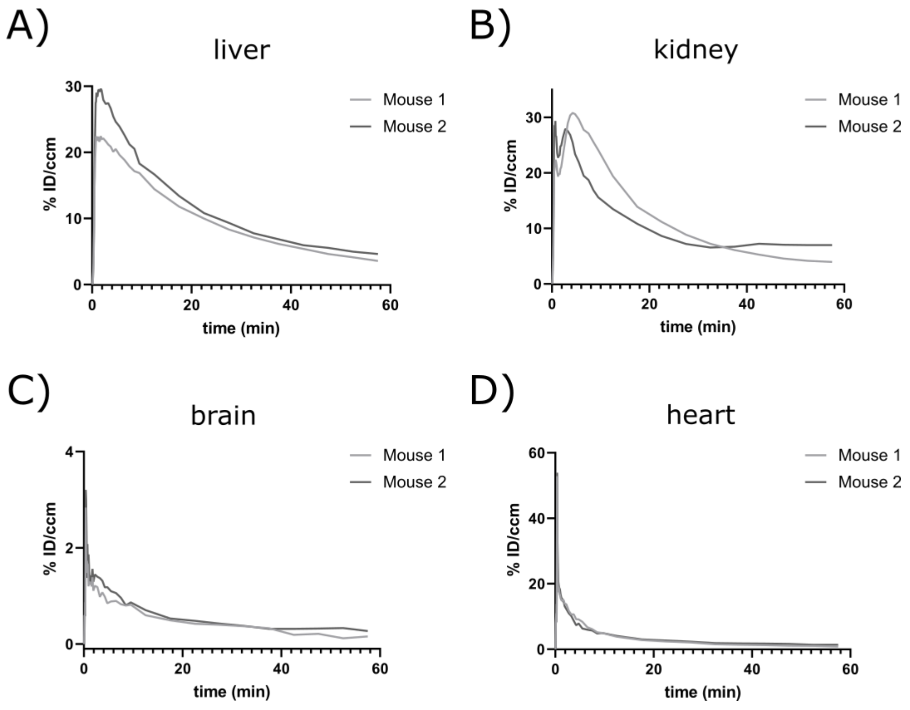

Supplementary Figure S4: TACs of liver, kidney, brain and heart for [^{18}F]*m*FBG.

Synthesis of precursor molecules

DABCO-guaninyl chloride (DCG)

DABCO-guaninyl chloride (DCG) was prepared in accordance with the literature published procedure.[1] 1,4-Diazabicyclo[2.2.2]octane (DABCO, 3.3 g, 29.4 mmol) and 2-amino-6-chloropurine (1.0 g, 5.8 mmol) were stirred together in anhydrous DMF (20 ml) for 1.5-3 hours at room temperature. The Resulting precipitate was collected by filtration and washed with a small quantity of warm DMF. The solid was dried at 70°C under high vacuum for 5 hours, affording the product salt as an off-white powder which was used without any further purification or analysis (1.62 g, 98% yield).

Synthesis of non-radioactive standard molecules:

Both 6-((4-fluorobenzyl)oxy)-9*H*-purin-2-amine (*p*FBG) and 6-((3-fluorobenzyl)oxy)-9*H*-purin-2-amine (*m*FBG) were synthesized via the same general procedure which was adapted from the available literature.[1]

General Procedure: Sodium hydride (60% in mineral oil) was suspended in DMSO (3 ml) a dry argon purged reaction flask at room temperature. A solution of fluorobenzyl alcohol in DMSO (1 ml) was added to the NaH suspension in slow dropwise fashion and the resulting mixture was allowed to stir at room temperature for 30 minutes. Solid DCG was added in portions to the reaction mixture and DMSO (1 ml) was used to wash the compound off the walls of the flask into the reaction mixture. The reaction was found (by TLC anaylsis) to run to completion within 30 minutes to an hour. When the reaction was deemed compete, it was quenched with saturated ammonium chloride solution (10 X the reaction volume) and the product was extracted with ethyl acetate. The organic fractions were pooled, dried with magnesium sulfate, and evaporated *in vacuo* to afford the crude product residue, which was later purified via flask chromatography (5% MeOH in DCM).

6-((4-fluorobenzyl)oxy)-9*H*-purin-2-amine (*p*FBG)

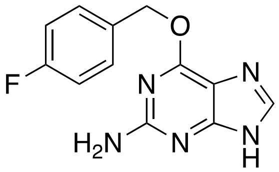

The synthesis of *p*FBG was carried out in accordance with the general procedure described above using sodium hydride (60% in mineral oil, 54 mg, 1.33 mmol), 4-fluorobenzyl alcohol (134 mg, 1.06 mmol), and DCG (250 mg, 0.89 mmol) in DMSO (5 ml total volume). Flask chromatography (5% MeOH in DCM) afforded the pure product (72 mg, 31%) and the analytical data was found to correspond exactly with the data published in literature.[1] MS (ESI): $[M]^+$ (theor.) = 259.09, Measured $[M+H]^+$ = 260.10; ^{1}H NMR (600 MHz, DMSO) δ: 12.40 (Brs, 1H, NH), 7.81 (Bs, 1H, Gua-ArH), 7.60 – 7.54 (m, 2H, Ar), 7.25 – 7.18 (m, 2H, Ar), 6.21 (Brs, 2H, NH_2), 5.47 (s, 2H, CH_2).

6-((3-fluorobenzyl)oxy)-9*H*-purin-2-amine (*m*FBG)

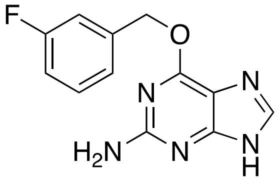

The synthesis of *m*FBG was carried out in accordance with the general procedure described above using sodium hydride (60% in mineral oil, 71 mg, 1.78 mmol), 3-fluorobenzyl alcohol (168 mg, 1.33 mmol), and DCG (250 mg, 0.89 mmol) in DMSO (5 ml total volume). Flask chromatography (5% MeOH in DCM) afforded the pure product (151 mg, 66%) and the analytical data was found to correspond exactly with the data published in literature.[1]

MS (ESI): $[M]^+$ (theor.) = 259.09, Measured $[M+H]^+$ = 260.10; ^{1}H NMR (600 MHz, DMSO) δ: 12.43 (Brs, 1H, NH), 7.83 (s, 1H, Gua-ArH), 7.44 (td, J = 8.0, 6.0 Hz, 1H, Ar), 7.36 – 7.31 (m, 2H, Ar), 7.20 – 7.14 (m, 1H, Ar), 6.22 (s, 2H, NH_2), 5.51 (s, 2H, CH_2).

N-(4-(((2-amino-9*H*-purin-6-yl)oxy)methyl)benzyl)-4-fluorobenzamide (FBBG)

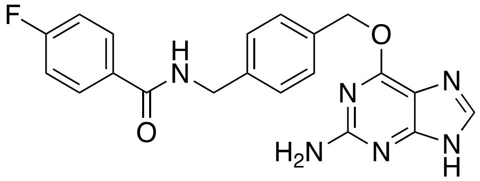

To a room temperature solution of 6-((4-(aminomethyl)benzyl)oxy)-7*H*-purin-2-amine (BG-NH_2, 20.0 mg, 74.0 µmol, AA Blocks, San Diego, CA, USA) and triethylamine (100 µl) in acetonitrile (1 ml) was added SFB (20.0 mg, 84.3 µmol, 1.14 eq; ABX, Radeberg, Germany). The mixture was stirred for 6 hours under argon, after which the solvent was removed under reduced pressure. The resulting residue was dissolved in a small amount of methanol and purified using silica gel column chromatography, using a gradient of 0-15 methanol in DCM. The resulting product was obtained as a white crystalline solid (25,97 mg, 80%). MS (ESI): $[M+H]^+$ (theor.) = 392.39, Measured 393.15; ^{1}H NMR (600 MHz, DMSO/D_2O 1:1). δ 8.00 – 7.93 (m, 3H), 7.59 (d, J = 7.8 Hz, 2H), 7.46 (d, J = 7.8 Hz, 2H), 7.34 (t, J = 8.8 Hz, 2H), 5.62 (s, 2H), 4.62 (s, 2H). (no exchangeable protons, referenced to dmso). ^{13}C NMR (151 MHz, DMSO) δ 172.81, 165.17, 163.90 (d, J = 248.4 Hz), 159.63, 139.49, 135.25, 130.79 (d, J = 3.0 Hz), 129.95 (d, J = 8.9 Hz), 128.58, 127.34, 115.24 (d, J = 21.6 Hz), 66.58, 42.50.

Supplementary References

1. Liu, X.; Zheng, Q.-H.; Hutchins, G.D.; Fei, X.; Erickson, L.C.; Miller, K.D.; Mock, B.H.; Glick-Wilson, B.E.; Winkle, W.L.; Lee Stone, K.; et al. A Convenient Procedure for the Synthesis of O6-Benzylguanine Derivatives by Phase Transfer Catalysis. *Synthetic Communications* **2003**, *33*, 941-952, doi:10.1081/SCC-120016358.
